# Universal correlation between *H*-linear magnetoresistance and *T*-linear resistivity in high-temperature superconductors

**DOI:** 10.1038/s41467-024-52564-3

**Published:** 2024-09-27

**Authors:** J. Ayres, M. Berben, C. Duffy, R. D. H. Hinlopen, Y.-T. Hsu, A. Cuoghi, M. Leroux, I. Gilmutdinov, M. Massoudzadegan, D. Vignolles, Y. Huang, T. Kondo, T. Takeuchi, S. Friedemann, A. Carrington, C. Proust, N. E. Hussey

**Affiliations:** 1 H. H. Wills Physics Laboratory, University of Bristol, Bristol, UK; 2 High Field Magnet Laboratory (HFML-EMFL) and Institute for Molecules and Materials, Radboud University, Nijmegen, Netherlands; 3 LNCMI-EMFL, CNRS UPR3228, Univ. Grenoble Alpes, Univ. Toulouse, INSA-T, Toulouse, France; 4 Max-Planck-Institute for the Structure and Dynamics of Materials, Hamburg, Germany; 5 Department of Physics, National Tsing Hua University, Hsinchu, Taiwan; 6 Van der Waals-Zeeman Institute, University of Amsterdam, Amsterdam, Netherlands; 7 Institute for Solid State Physics, University of Tokyo, Kashiwa, Japan; 8 Toyota Technological Institute, Nagoya, 468-8511 Japan

**Keywords:** Electronic properties and materials, Superconducting properties and materials

## Abstract

The signature feature of the ‘strange metal’ state of high-*T*_*c*_ cuprates—its linear-in-temperature resistivity—has a coefficient *α*_1_ that correlates with *T*_*c*_, as expected were *α*_1_ derived from scattering off the same bosonic fluctuations that mediate pairing. Recently, an anomalous linear-in-field magnetoresistance (=*γ*_1_*H*) has also been observed, but only over a narrow doping range, leaving its relation to the strange metal state and to the superconductivity unclear. Here, we report in-plane magnetoresistance measurements on three hole-doped cuprate families spanning a wide range of temperatures, magnetic field strengths and doping. In contrast to expectations from Boltzmann transport theory, *γ*_1_ is found to correlate universally with *α*_1_. A phenomenological model incorporating real-space inhomogeneity is proposed to explain this correlation. Within this picture, superconductivity in hole-doped cuprates is governed not by the strength of quasiparticle interactions with a bosonic bath, but by the concentration of strange metallic carriers.

## Abstract (web summary)

Understanding “strange metal" behavior in high-temperature superconductors remains an open problem. Here the authors report a correlation between linear-in-magnetic-field magnetoresistance and linear-in-temperature resistivity in several hole-doped cuprate families and discuss its possible implications for superconductivity.

## Introduction

The temperature (*T*) and magnetic field (*H*) dependence of the electrical resistivity *ρ* of a metal contains a wealth of information on the dominant interactions that scatter the conduction electrons and thus can mediate superconducting (SC) pairing. In conventional metals, the *T*-linear electron-phonon (e-ph) scattering rate *ℏ*/*τ*_*ph*_ = 2 *πλ*_*tr*_*k*_*B*_*T* where *ℏ* is Planck’s constant, *k*_*B*_ Boltzmann’s constant and *λ*_*tr*_ is the e-ph coupling constant for transport. *λ*_*tr*_ is closely related to *λ*, the e-ph coupling strength used in the determination of *T*_*c*_ in BCS superconductors via the McMillan formula^1^; a correspondence which underlies the old adage ‘good metals make bad superconductors’.

This correlation between *λ*_*tr*_ and *T*_*c*_ contrasts in an interesting way with what is found in certain quantum critical metals that also superconduct. In these materials, a fan-like region of *T*-linear resistivity, emanating from a quantum critical point (QCP), is attributed to scattering off critical fluctuations of the underlying order^2,3^ that is strong enough to destroy the Fermi-liquid (FL) ground state. Empirically, both *α*_1_ (the coefficient of the *T*-linear resistivity) and the extracted scattering rate *ℏ*/*τ* ~ *ζk*_*B*_*T* (1 ≤ *ζ* ≤ *π*) are approximately constant within the quantum critical fan^3,4^ and as such, do not correlate with *T*_*c*_. This lack of correlation supports the notion that inelastic scattering within the fan is bounded to a maximal equilibration rate for charge carriers known as the Planckian limit^5,6^. The notion of Planckian dissipation is now associated with a broad class of strongly interacting materials, including high-*T*_*c*_ cuprates^5,7,8^, ultracold atoms^9^ and twisted bilayer graphene^10^, motivating the search for a unified explanation with^11,12^ or without well-defined quasiparticles^13,14^.

In overdoped cuprates, the low-*T* in-plane resistivity is empirically well described by *ρ*_*ab*_(*T*) = *ρ*_0_ + *α*_1_*T* + *βT*^2^ where *α*_1_ is finite not at a singular point but over an extended doping range—from just beyond optimal doping to the edge of the SC dome^7,15^. Such extended *T*-linearity to the lowest *T* is suggestive of a quantum critical *phase*^14,16^ and is a defining characteristic of the cuprate ‘strange metal’ regime^17^. In marked contrast to what is found in quantum critical metals, *α*_1_ in overdoped cuprates is correlated with both *T*_*c*_^7,15^ and the superfluid density *n*_*s*_^17^. At first sight, these correlations suggest an analogy with conventional BCS superconductors, with spin^18^ or charge^19^ fluctuations possibly playing the role of the scattering boson for transport and Cooper pair formation alike. The existence of an interacting boson of variable strength (i.e., variation in *α*_1_), as well as the presence of a *T*^2^ scattering rate, however, sit at odds with the notion of universal Planckian dissipation^8^.

In order to gain further insights into the nature of the strange metal (SM) regime in cuprates, attention has switched to the magnetoresistance (MR) response at high fields (20 T ≤ *μ*_0_*H* ≤ 90 T), specifically the robust *H*-linear MR observed in both electron-^20^ and hole-doped cuprates^21–23^. To date, these high-field studies have tended to focus on a rather narrow, isolated region of their respective phase diagrams and as such, any attempt to find a relation between the *H*-linear MR and *T*-linear resistivity has been speculative. To address this, we have carried out a comprehensive high-field MR study on three hole-doped cuprate families: (Pb/La)-doped Bi_2_Sr_2_CuO_6+*δ*_ (Bi2201), Tl_2_Ba_2_CuO_6+*δ*_ (Tl2201) and La_2−*x*_Sr_*x*_CuO_4_ (LSCO) for which the correlation between *α*_1_ and *T*_*c*_ is well established^7,24,25^. Our study reveals a striking and robust correlation between *α*_1_ and *γ*_1_, the slope of the high-field *H*-linear MR. A simple, phenomenological model is proposed that is able to account both for the (*T* + *T*^2^) form of the resistivity and for the non-trivial correlation between *α*_1_ and *γ*_1_. When combined with the correlation between *α*_1_, *T*_*c*_ and *n*_*s*_, we conclude that weakening superconductivity in overdoped cuprates is governed not by a decreasing strength of quasiparticle interactions, but by a reduction in the number of strange metallic (possibly Planckian) carriers.

## Results

**Fig. 1:**
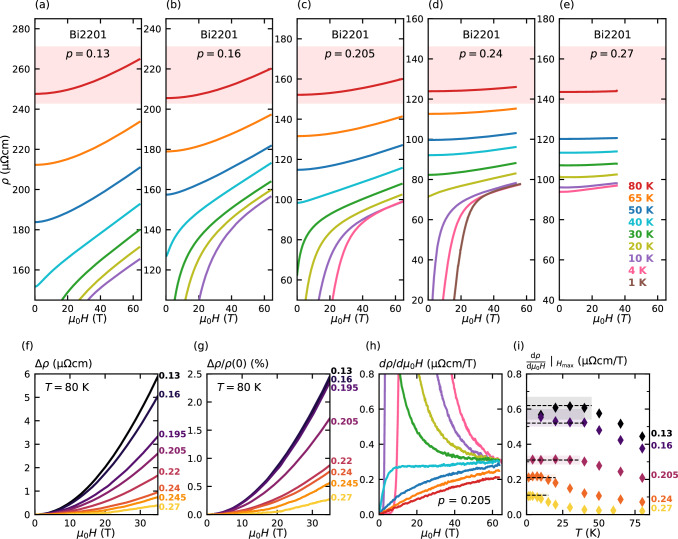
Doping dependence of the transverse MR in Bi2201. **a**–**e** *ρ*(*H*, *T*) of selected Bi2201 crystals over the doping range 0.13 ≤ *p* ≤ 0.27. With increasing *p*, a marked decrease in the magnitude of the MR (at a constant temperature) is observed, as highlighted by the red shaded regions for *T* = 80 K. **f** Δ*ρ*(*H*, 80 K) curves for 8 different dopings. **g** Corresponding Δ*ρ*/*ρ*(0) curves. For *p* > p* ~ 0.2, the magnitude of Δ*ρ*/*ρ*(0) drops with increasing *p*, revealing that within the SM regime (p* < *p* < 0.27), the order-of-magnitude enhancement in the MR is not a result of an increase in *ρ*(0, *T*) (e.g. due to a reduced carrier density). **h** The derivative of the MR at *p* = 0.205 illustrating the tendency at all temperatures to *H*-linearity at high fields. The line colours are the same as those used in (**a**)–(**e**). **i** *T*-dependence of d*ρ*/d*μ*_0_*H* at the highest measured field range (specifically the last 3 T of each field sweep) for the MR curves displayed in (**a**)–(**e**). For all dopings, d*ρ*/d${\mu }_{0}H{| }_{{H}_{\max }}$ saturates at low *T* at a value (= *γ*_1_) denoted by horizontal dashed lines. Shaded regions reflect the uncertainty in *γ*_1_ for each data set. Estimates for *γ*_1_ obtained from field sweeps for which the *H*-linear regime could not be reached are not shown. For the optimally-doped sample (*p* = 0.16), the upturn in d*ρ*/d${\mu }_{0}H{| }_{{H}_{\max }}$ at the lowest *T* is due to paraconductivity effects. The origin of the low-*T* downturn in d*ρ*/d${\mu }_{0}H{| }_{{H}_{\max }}$ for *p* = 0.13 is unknown. Nevertheless, an estimate for *γ*_1_ can still be made, albeit with greater uncertainty.

Panels (a–e) of Fig. 1 show *ρ*(*H*, *T*) for a representative set of Bi2201 single crystals over a wide doping range that incorporates both the pseudogap (0.13 ≤ *p* < p* ~ 0.20) and strange metal (p* < *p* ≤ 0.27) regimes. (In all measurements shown here, the current *I* is applied within the CuO_2_ plane and **H** ∥*c*). In highly doped samples with a low *T*_*c*_, the crossover from quadratic to linear MR is clearly visible within the accessible field range. At lower dopings where *T*_*c*_ is elevated, the low-*T* behaviour is obscured by superconductivity and only *H*-linearity is observed at high fields. At higher *T*, the MR is predominantly quadratic but approaches linearity at high fields with a coefficient in agreement with the data reported over a much more limited field and doping range in ref. ^22^. In all cases, *H*-linearity is asymptotically approached at the highest fields.

The red shaded regions of panels (a–e) highlight the doping evolution of the MR at a fixed temperature *T* = 80 K that in all cases lies well above the temperature below which paraconductivity contributions from SC fluctuations become apparent. Noting that the absolute span in *ρ* is the same (140 *μ*Ωcm) in all panels, it is evident that the magnitude of the MR decreases monotonically with increasing *p*. The overall trend is summarized in Fig. 1f where Δ*ρ*(*H*, 80 K) = (*ρ*(*H*, 80 K) –*ρ*(0, 80 K)) is plotted for 8 different dopings. *ρ*(0, 80 K) itself decreases roughly by a factor of 2 across the series, reflecting changes in the scattering rate and/or the carrier density. Δ*ρ*(*H*, 80 K), by contrast, decreases by more than one order of magnitude. The corresponding Δ*ρ*(*H*, 80 K)/*ρ*(0, 80 K) values are plotted in Fig. 1g. Intriguingly, for the three samples in the pseudogap regime with *p* < p* (~0.2), Δ*ρ*(*H*, 80 K)/*ρ*(0, 80 K) is found to be independent of doping. The same is also seen in LSCO below p*—see Supplementary Fig. 3c. This implies that the increase in Δ*ρ* below p* is a direct consequence of the increase in *ρ*(0) with underdoping, presumably due to the loss of electronic states once the pseudogap opens. For higher dopings, by contrast, even the fractional change in MR is found to vary strongly with *p*, decreasing by one order of magnitude between *p* = 0.205 and 0.27 (see Fig. 1g).

At high *H*/*T* (below *T*_*c*_ in most samples) and for all dopings, the MR asymptotically approaches *H*-linearity with a *T*-independent slope *γ*_1_, as shown in ref. ^22^ and illustrated for *p* = 0.205 in Fig. 1h. In order to obtain estimates for *γ*_1_(*p*), we study the low-*T* limit of the highest field data (where the normal state can be accessed). A summary of the results is shown in Fig. 1i where the *T*-dependence of d*ρ*/d${\mu }_{0}H{| }_{{H}_{\max }}$—the MR slope at the highest measured field range (i.e. over the last 3 T of each field sweep)—is plotted for all the samples whose raw MR data are included in panels (a–e). In agreement with previous measurements on LSCO^21^, d*ρ*/d${\mu }_{0}H{| }_{{H}_{\max }}$ is found to increase with decreasing *T* and to saturate at a constant value (=*γ*_1_) at low *T*. As a function of doping, *γ*_1_(*p*) exhibits a monotonic decrease, consistent with our expectations from Fig. 1g.

Two further studies were conducted: one on a single crystal of overdoped Tl2201 (*T*_*c*_ = 35 K at ambient pressure) as a function of hydrostatic pressure and one on a series of LSCO single crystals, the data for which are reported in Supplementary Figs. 2 and 3, respectively. The *T*_*c*_ (and inferred doping) of Tl2201 can be tuned appreciably with the application of modest pressures^26^. A gradual decrease in *γ*_1_ is observed with increasing pressure, consistent with the correlation between *γ*_1_ and *T*_*c*_ (or *p*) seen in Bi2201 beyond p*. As reported in ref. ^21^, LSCO near p* (*x* = 0.19) also exhibits *H*-linear MR at the highest fields. Here, the MR of two LSCO samples with *x* = *p* = 0.20 and 0.23 up to 35 T has been measured and pulsed-field data reported in ref. ^7^ for *p* = 0.17 and 0.23 reanalysed. The field derivatives show clearly that, just as in Bi2201 and Tl2201, the MR becomes *H*-linear at high *H*/*T* values.

**Fig. 2:**
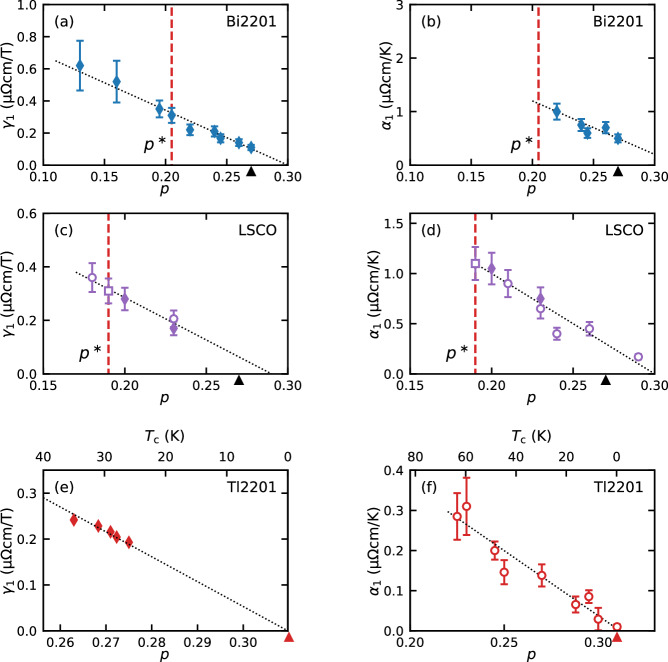
Universal correlation between *γ*_1_ and *α*_1_ in overdoped cuprates. Doping dependence of *γ*_1_—the high *H*/*T* limiting value of the MR—and *α*_1_—the low-*T* *T*-linear coefficient of the resistivity—for (**a**, **b**) Bi2201, (**c**, **d**) LSCO and (**e**, **f**) Tl2201, respectively. Diamonds indicate results obtained as part of this high-field study, while circles and squares indicate results obtained from the literature. Circles and squares in (**c**) and (**d**) are taken from refs. ^7^, ^21^, respectively. Circles in (**f**) are from ref. ^24^. Note that *α*_1_ is plotted only for samples with *p* > p*. In **e**, *γ*_1_ is plotted vs. *p* as determined from the *T*_*c*_ value at each pressure using the correlation reported in ref. ^25^. Error bars on points marked as solid diamonds are due to geometric uncertainty in the absolute value of the resistivity and necessary extrapolations. Error bars on the open squares and circles are taken from the relevant source, if provided, or assumed to be 15% (typical uncertainty in the geometrical ratios) if not. In (**e**), no random uncertainty in contact geometry is present due to a single sample being measured but a systematic uncertainty of 20% is included. All dotted lines are guides to the eye. Arrowheads on the doping axes indicate *p*_*sc*_ for the respective material.

The results for all three families are summarised in Fig. 2; the left and right panels showing, respectively, the doping or pressure evolution of *γ*_1_ and *α*_1_. (Although an equivalence between pressure and hole doping in Tl2201 has not yet been definitively demonstrated, we adopt here the dependence of *T*_*c*_ on *p* reported in ref. ^25^ to arrive at the same conclusion). Despite the very different Fermi surface topologies, degree of electronic inhomogeneity and electron mobility across the three families, both *α*_1_ and *γ*_1_ exhibit a clear linear doping dependence and extrapolate to zero at the same doping concentration in all cases, implying a direct and robust correlation between the two. That both coefficients extrapolate to zero at a doping level *p* ≈ 0.3 and positively correlate with *T*_*c*_ throughout the SM regime also implies that the emergence of *H*-linear MR and *T*-linear resistivity is closely tied to the onset of superconductivity. While previous studies^20–22^ have hinted at a trend in *γ*_1_, the doping ranges in each case were too narrow (Δ*p* ≤ 0.03) to make any definitive claims.

## Discussion

In order to understand why these correlations are non-trivial, let us first consider the MR response of a FL described by Boltzmann transport theory. Being an even function of *H*, the MR is always quadratic at the lowest field strengths, though it is the cyclotron frequency *ω*_*c*_ (∝*H*), not *τ*, that sets the field dependence of Δ*ρ*(*H*). Its magnitude, on the other hand, is set primarily by the product *ω*_*c*_*τ* and its variation (anisotropy) over the Fermi surface. (Recall that MR vanishes in a perfectly isotropic metal^27^). Provided that this anisotropy does not vary significantly with temperature, the *T*-dependence of Δ*ρ*(*H*) will be determined largely by that of *ρ*(*T*), as expressed through Kohler’s rule: Δ*ρ*/*ρ*(0, *T*) = *f*(*H*/*ρ*(0, *T*)). With increasing field strength, Δ*ρ*(*H*) will pass through an inflection point before saturating once multiple cyclotron orbits have washed out all manifestations of anisotropy. Hence, unless the anisotropy itself is extreme^28^ or multiband effects are involved^29^, no extended region of *H*-linear MR is expected.

With regards to the doping dependence, a decreasing magnitude of the MR due to a monotonic reduction in *ω*_*c*_*τ* at low-*T* as the system is doped away from the Mott insulating state (and thus becomes increasingly more metallic) seems unlikely; there is little variation in the residual resistivity *ρ*_0_ within this doping range and no indication, e.g. from existing specific heat measurements^30,31^, for a large change in m*. Moreover, the magnitude of *γ*_1_ in Bi2201 and Tl2201 is essentially the same, despite their *ρ*_0_ values differing by almost one order of magnitude.

The alternative explanation—a reduction in the effective anisotropy of *ω*_*c*_*τ* with increasing *p*—could in principle apply to LSCO. Strong in-plane anisotropy in the elastic mean-free-path *ℓ*_0_(*ϕ*) has been deduced at a doping close to where the Fermi level crosses the van Hove singularity (vHs)^32^ and shown to generate a broad regime of *H*-linear MR of the right order of magnitude^23^. Moreover, beyond the edge of the SC dome, this anisotropy in *ℓ*_0_(*ϕ*) is known to be much reduced^33^. As shown in Supplementary Fig. 4, however, incorporating the known Fermi surface geometry and a form of *ℓ*_0_(*ϕ*) that tracks the anisotropy in the density of states into the Boltzmann equation, we find that Δ*ρ*/*ρ*(0) and *γ*_1_ are essentially doping independent across the SM regime, in marked contrast with experimental findings.

More constraining is the fact that in Bi2201, the vHs crossing point is located close to *p* ~ 0.27^34,35^, i.e. where the MR is smallest, while in Tl2201, it is believed to be located at a doping level (*p* ~ 0.54) that is far higher than those studied here^25^. Hence, in both Bi2201 and Tl2201, one should expect anisotropy in *ℓ*_0_(*ϕ*) and the resultant MR to grow with increasing *p* (as confirmed in the simulations for Bi2201 plotted in Supplementary Fig. 5). Thus, given what is known about these three distinct families, the ubiquitous and marked decrease in *γ*_1_ across the SM regime appears difficult to reconcile within any viable Boltzmann framework, even one incorporating a very specific combination of Fermiology and anisotropic scattering.

There are several other known mechanisms for generating a *H*-linear MR in metals, in particular, those that incorporate a random distribution in carrier density and mobility. As we discuss in the Supplementary Information Section IV, however, none of these seem capable of explaining the key observations: the *H*/*T* scaling of the MR in the presence of a large *ρ*_0_, the correlation between *α*_1_ and *γ*_1_ and the presence of the *T*^2^ component in *ρ*(*T*) in a consistent manner. What is both intriguing and constraining here is the ubiquity of the *H*-linear MR (in multiple families with different Fermiologies), its magnitude, form and *T*-dependence and the aforementioned correlations (with *α*_1_ and *T*_*c*_).

One of the most striking features of the MR response in overdoped cuprates is its empirical adherence to the quadrature expression: *ρ*(*H*, *T*) = ${{\mathcal{F}}}(T)+\sqrt{{(\alpha {k}_{B}T)}^{2}+{(\gamma {\mu }_{0}H)}^{2}}$ ^36^, where *α* (*γ*) are the *T*- (*H*-)linear coefficients within the quadrature expression, respectively, and ${{\mathcal{F}}}(T) \sim {\rho }_{0}+\beta {T}^{2}$ ^22^. (Note here that the entire field response is encapsulated in the quadrature expression; the ${{\mathcal{F}}}(T)$ component itself possessing negligible MR.) An example of the applicability of this expression is shown for Tl2201 in Supplementary Fig. 2 (dashed lines). Such a decomposition of *ρ*(*H*, *T*) may signify either the presence of two independent inelastic scattering rates (one *T*-linear, one quadratic) or two resistive channels coupled in series. For the former, these rates should combine with the elastic (impurity) scattering to generate a total scattering rate whose overall *T*-dependence is also reflected in the MR, an outcome that is inconsistent with the strict adherence to the quadrature expression (in which only the *T*-linear component of *ρ*(*T*) appears in the MR). Secondly, the large value of *ρ*_0_ in Bi2201 and the lack of sufficient *k*-space anisotropy in the mean-free-path *ℓ* in Tl2201 at low *T*^37^ ensure that any MR calculated using Boltzmann transport theory and the known parameterization for each system is at least one order of magnitude smaller than what is seen in experiment. Given these empirical facts, we proceed by assuming that *ρ*(*H*, *T*) is composed of two distinct sectors (phases) separated in real space, only one of which contributes significantly to the MR.

Overdoped cuprates have long been considered as systems exhibiting microscopic phase separation and granular superconductivity^38–40^. In Bi2201, for example, real-space patchiness has been imaged directly by STM for all dopings within the SM regime^41^. Patchiness has also been inferred for strongly overdoped LSCO^42^. Guided by this and the tendency for the magnetotransport to interpolate smoothly from pure FL-like behaviour (quadratic resistivity and small MR) at high dopings to pure SM behaviour (*T*-linear resistivity and large MR) near p*, we apply effective medium theory (for which the granularity of the model is scale-invariant) to a network of distinct FL and non-FL patches.

**Fig. 3:**
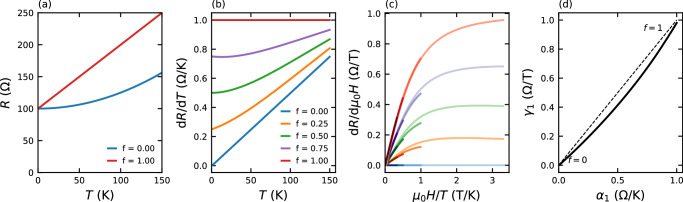
Modelling the doping dependence of the MR with effective medium theory. A fraction *f* of the sample is assumed to have a zero-field *T*-linear resistance and quadrature MR: ${R}_{SM}=100+\sqrt{{T}^{2}+{({\mu }_{0}H)}^{2}}$. The remainder of the sample is assigned a FL resistance with no MR: *R*_*FL*_(*T*, *B*) = 100 + *T*^2^. The macroscopic resistance of the sample is then computed for various values of *f* using effective medium theory as described in ref. ^43^. **a** Resistance *R* of the sample in limiting cases where *f* = 0, 1. **b** Temperature derivative of the resistance *dR*/*dT* for different values of *f*. A systematic increase in *α*_1_, the low-*T* *T*-linear coefficient of the resistance, is found with increasing *f*. **c** Field derivative of the sample resistance *dR*/*dH* plotted against *H*/*T*. With decreasing *f*, the quadrature MR intrinsic to the strange metallic part of the sample is diluted by an increasing fraction of the FL component. The scaling is reasonably well maintained for intermediate *f*. **d** The *T*-linear coefficient of the resistance *α*_1_ is seen to correlate with *γ*_1_—the *H*-linear slope of the high field MR—with both growing monotonically with increasing *f*.

According to this picture, a fraction *f* of the sample is assigned a strange metallic *T*-linear resistance while the remainder is assigned a FL quadratic resistance. The resistance of the sample for the two limiting cases where *f* = 0, 1 are shown in Fig. 3a. For intermediate *f*, the macroscopic resistance is computed following^43^. This is already sufficient to reproduce a sample resistance that at low *T* has the observed *α*_1_*T* + *βT*^2^ form with *α*_1_ smoothly increasing as a function of *f* (Fig. 3b). If one further assumes that the strange metal patches also have an intrinsic MR than scales with *H*/*T* (justified by the observed adherence to the quadrature form) and that the FL-like resistors have a negligible MR (in accordance with expectations from Boltzmann theory), one obtains an MR that retains *H*/*T* scaling reasonably well for all values of *f* (Fig. 3c) with a high-field *H*-linear slope *γ*_1_ that correlates with *α*_1_ (Fig. 3d).

Our phenomenological model, though overly simplistic, does appear to capture most, if not all, of the key observations: the low-*T* form of the resistivity (*ρ*_0_ + *α*_1_*T* + *βT*^2^), the quadrature form of the MR and associated *H*/*T* scaling as well as the correlation between *γ*_1_ and *α*_1_ self-consistently. While the distinct patchwork picture introduced here is probably not by itself sufficient, we expect that real-space inhomogeneity of the type highlighted in this study will be an essential ingredient of any subsequent microscopic model. Distributed networks of metallic components have previously been invoked to explain the MR of both pnictides^44^ (which also scales with *H*/*T*) and cuprates^45^ at singular dopings where the resistivity is purely *T*-linear. 2D random resistor networks are capable of producing non-saturating *H*-linear MR by incorporating continuous resistivity or mobility disorder^46^, while inhomogeneity in the carrier density can generate a similar MR response^47^. Moreover, an extension of the resistor network model to 3D returns a *H*-linear MR in longitudinal fields^48^ as observed in both Tl2201 and Bi2201 for **H** ∥*ab* and for which an explanation is currently lacking^22^. One salient property of this variety of model is that the resultant behaviour is insensitive to details of the Fermiology that (within a Boltzmann framework) should distinguish the various materials studied but appear not to. It remains a challenge, however, for such models to reproduce the *H*/*T* scaling, a feature that appears to set this particular MR response apart from all others.

In closing, our study has revealed a robust correlation between the coefficient *α*_1_ of the *T*-linear resistivity and the *H*-linear slope of the high-field MR in three distinct families of hole-doped cuprates. It has been argued that such a universal correlation cannot be reconciled with any simple reformulation of Boltzmann transport theory. Although the present two-component model cannot account for all of the in-plane transport properties of overdoped cuprates^25^, it nevertheless provides a basic framework that captures much of the essential phenomenology on which a more complete microscopic model may be built. According to this model, the doping dependences of *α*_1_ and *γ*_1_ arise due to a change in the fraction of SM carriers across the overdoped regime. As mentioned previously, the doping dependence of the *T*-linear resistivity seemingly sits at odds with an origin rooted in dissipation at a Planckian limit. Nevertheless, if the Planckian scattering is confined to a subset of the carriers, as proposed here, its variation with doping might still be reconciled with the notion of a Planckian bound^8^. The fact that the fall in superfluid density with overdoping also tracks the decrease in the number of SM carriers is intriguing, as it implies that the SC condensate emerges predominantly, if not uniquely, from the *♯*3.7 non-FL^49^ or non-itinerant^39^ sector. Consequently, the suppression of the condensate is seen to be linked directly to the demise of the strange metal and not to a reduction in pairing strength and any associated (disorder-induced) pair-breaking. The reason why *α*_1_, *γ*_1_ and *T*_*c*_ are correlated across the SM regime then becomes apparent.

## Methods

High quality Bi2201 and LSCO crystals were grown in floating zone furnaces at 3 different sites. To cover much of the phase diagram, some of the Bi2201 crystals were doped with La and Pb, resulting in the chemical formula: Bi_2+*z*−*y*_Pb_*y*_Sr_2−*x*−*z*_La_*x*_CuO_6+*δ*_. The Tl2201 crystal studied here was synthesized using a self-flux technique^50^. The hole doping for Bi2201 was estimated from the resistively-measured *T*_*c*_ using the Presland relation^51^: 1–*T*_*c*_/${T}_{c}^{\max }$ = 82.6(*p* – 0.16)^2^ with ${T}_{c}^{\max }$ = 35 K. This relation was recently demonstrated to hold well in Bi2201^35^. For LSCO, the doping was estimated from the measured *T*_*c*_ using the same parabolic relation with ${T}_{c}^{\max }$ = 38 K and found to match closely the Sr content of each crystal. The crystallographic axes of LSCO were oriented with a Laue camera. Typical sample dimensions were 1000 × 250 × 10 *μ*m^3^ for Bi2201 and Tl2201 and 1500 × 250 × 50 *μ*m^3^ for LSCO. The *ρ*(0, *T*) curves of all the MR samples are shown in Supplementary Fig. 1.

The MR was measured in DC and pulsed magnets up to 35 T and 70 T, respectively with the current *I* applied in-plane and **H** ∥*c*. For the pulsed field measurements, samples and wires were fully covered in GE varnish and/or vacuum grease to reduce vibration. To increase the measurement signal, each Bi2201 crystal was mechanically thinned to a thickness of 2–10 *μ*m, resulting in sample resistances of ~1 Ω, i.e. comparable to the resistance of the current contacts. At each measurement temperature, the MR curves were recorded for both polarities of the magnetic field. For Tl2201, a single crystal with an ambient pressure *T*_*c*_ = 35 K was selected and prepared for transport measurements under the application of hydrostatic pressure using a piston cylinder cell. The sample was oriented on a feed-through such that the magnetic field **H** ∥*c*. Daphne 7373 oil was used as a pressure transmitting medium as it is known to remain hydrostatic at room temperature (the temperature at which pressure was applied) up to 2.2 GPa^52^, beyond the pressures applied in this work.

## Supplementary information

Supplementary Information

Peer Review File

## Data Availability

The data that support the findings of this study are available at the University of Bristol data repository, data.bris, at 10.5523/bris.2g87jr92yk2v72t1wz0ynjcsts. Other material is available from the corresponding authors upon request.
