# Supplementary material for: Universal correlation between H-linear magnetoresistance and T-linear resistivity in high-temperature superconductors
Source: Nat Commun. 2024 Sep 27;15:8406. doi: 10.1038/s41467-024-52564-3 (PMC11436940; doi:10.1038/s41467-024-52564-3)
Supplement: Supplementary file 1 — Supplementary Information [file 41467_2024_52564_MOESM1_ESM.pdf]

Universal correlation between H-linear magnetoresistance and T-linear resistivity in high-temperature superconductors Supplementary Information

J. Ayres,[1,*] M. Berben,[2,*] C. Duffy,[2,3] R. D. H. Hinlopen,[1,4] Y.-T. Hsu,[2,5] A. Cuoghi,[2] M. Leroux,[6] I. Gilmutdinov,[6] M. Massoudzadegan,[6] D. Vignolles,[6] Y. Huang,[7] T. Kondo,[8] T. Takeuchi,[9] S. Friedemann,[1] A. Carrington,[1] C. Proust,[6] and N. E. Hussey[1,2]

[1]*H. H. Wills Physics Laboratory, University of Bristol, Bristol, United Kingdom*

[2]*High Field Magnet Laboratory (HFML-EMFL) and Institute for Molecules and Materials, Radboud University, Nijmegen, Netherlands*

[3]*LNCMI-EMFL, CNRS UPR3228, Univ. Grenoble Alpes, Univ. Toulouse, INSA-T, Toulouse, France*

[4]*Max-Planck-Institute for the Structure and Dynamics of Materials, Hamburg, Germany*

[5]*Department of Physics, National Tsing Hua University, Hsinchu, Taiwan*

[6]*LNCMI-EMFL, CNRS UPR3228, Univ. Grenoble Alpes, Univ. Toulouse, INSA-T, Grenoble and Toulouse, France*

[7]*Van der Waals-Zeeman Institute, University of Amsterdam, Amsterdam, Netherlands*

[8]*Institute for Solid State Physics, University of Tokyo, Kashiwa, Japan*

[9]*Toyota Technological Institute, Nagoya 468-8511, Japan*

(Dated: August 16, 2024)

* These two authors contributed equally

I. ZERO-FIELD RESISTIVITY OF ALL SAMPLES UNDER STUDY

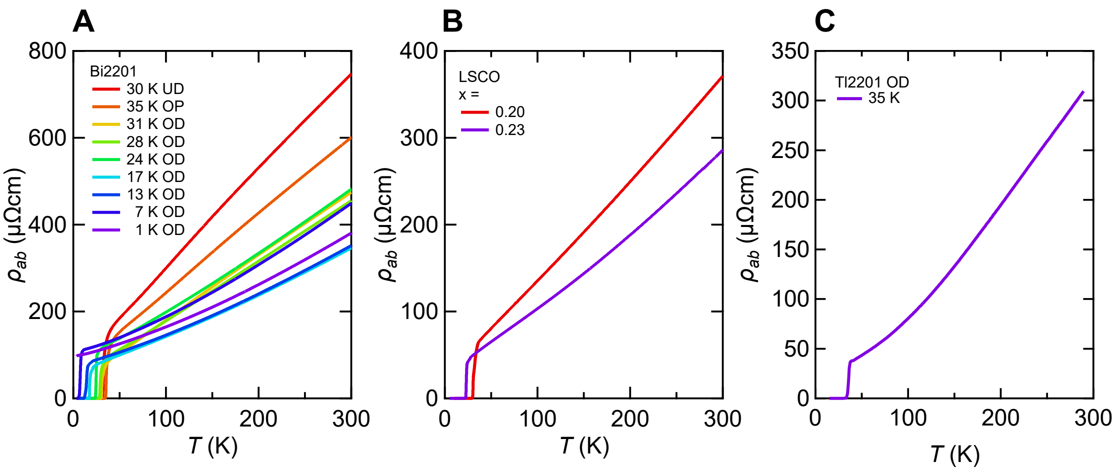

Supplementary Figure 1. Zero-field resistivity curves for the (a) Bi2201, (b) LSCO and (c) Tl2201 samples whose magnetoresistance was investigated as part of this study. The error in the absolute values of $\rho(0, T)$ (due to geometrical uncertainties) is estimated to be $\sim$ 15%.

II. MAGNETORESISTANCE OF OVERDOPED Tl2201 UNDER PRESSURE

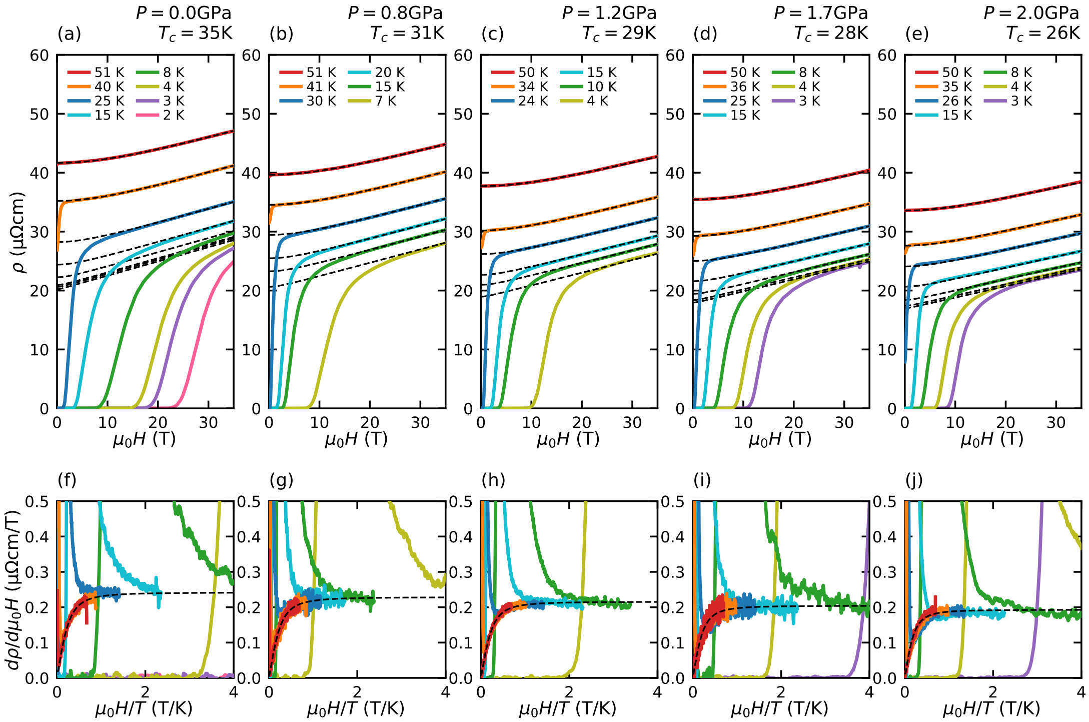

Supplementary Figure 2. (a)-(e) Evolution with pressure of $\rho(H,T)$ of the Tl2201 single crystal (ambient pressure $T_c = 35$K). Dashed lines are simultaneous fits to the expression $\rho(H,T) = \mathcal{F}(T) + \sqrt{(\alpha k_B T)^2 + (\gamma\mu_0 H)^2}$ where $\mathcal{F}(T) \sim \rho_0 + AT^2$. (f)-(i) Derivatives $\mathrm{d}\rho/\mathrm{d}(\mu_0 H)$ of the MR presented in panels (a)-(e). The derivatives presented in panels (f)-(i) are plotted against H/T. The dashed lines are derived from a single fit to the full set of MR derivatives (in the normal state) to the above expression. γ_1 – the limiting low-T, high-H slope of the H-linear MR – can be read off from the plateau region of each dashed line fit.

III. QUADRATURE SCALING OF THE MR IN OVERDOPED LSCO ($x = 0.23$)

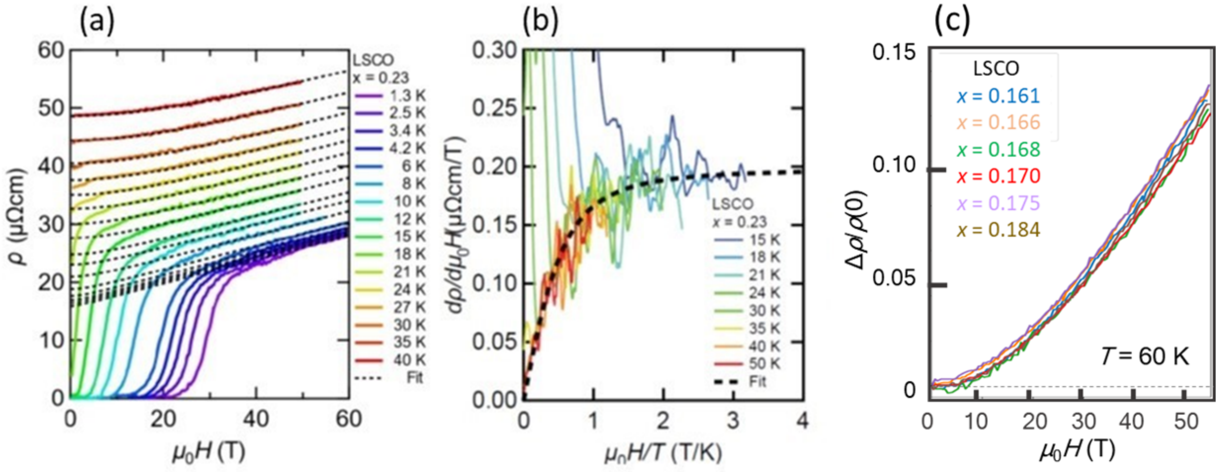

Supplementary Figure 3. (a) Reproduction of the set of MR curves for LSCO $x = 0.23$ reported in Ref. [3]. Dashed lines are fits to the quadrature magnetoresistance expression $\rho(H,T) = \mathcal{F}(T) + \sqrt{(\alpha k_B T)^2 + (\gamma \mu_0 H)^2}$. (b) Corresponding plot of $\mathrm{d}\rho/\mathrm{d}\mu_0 H$ vs. H/T indicating that the form of the MR does indeed follow H/T scaling. Dashed line is the fit to the derivative of aforementioned quadrature expression. (c) $\Delta\rho(H,\, 60\mathrm{K})/\rho(0,\, 60\mathrm{K})$ curves generated from the published high-field MR data for LSCO below p^* ($0.16 \leq x \leq 0.185$) by Giraldo-Gallo *et al.* [9]. Note that there is no 60 K data for the $x = 0.19$ sample shown in Ref. [9].

IV. ORIGINS OF H-LINEAR MR IN METALS

The observation of a linear-in-field magnetoresistance is, of course, not unique to cuprates and there are numerous models capable of reproducing a linear MR that have found success in their application to other systems. We will first outline those well known mechanisms that we believe are not at all applicable to cuprates before discussing the merits and shortcomings of other mechanisms that might be of relevance in more detail.

As they pertain to overdoped cuprates, the following is a non-exhaustive list of potential mechanisms that we believe can be excluded:

- Quantum magnetoresistance [4, 5] – the generation of a linear MR upon reaching the quantum limit, a regime made accessible in elemental Bi, for example, by the low carrier density and effective mass ($m^* \sim 10^{-2} m_e$) and high mobility. The carrier density and effective mass of cuprates preclude this possibility. H-linearity is observed in Bi2201 and Tl2201 at fields as low as 10 T which (given the large scattering rate of Bi2201 in particular) is well within the low field limit.

- Zeeman splitting – suggested to be relevant to pnictides [8] and cuprates [9, 10]. While the observed insensitivity of both the form and magnitude of the MR to field orientation in Bi2201 and Tl2201 [11] is consistent with such a scenario, the situation in LSCO [12, 13] is not.

- Magnetic breakdown [14] – The cuprates are single banded systems. There is no gap (at all or of an appropriate size) across which magnetic breakdown can occur.

- Sharp Fermi surface corners [6] – although relevant to various density-wave materials [7], the FS of overdoped Bi2201, Tl2201 in particular are believed to be devoid of sharp corners. LSCO is more anisotropic and whilst sharp features might be present close to the van Hove singularity (re-addressed later), they are not universally present.

Other explanations can be categorised into those based on reciprocal-space and real-space phenomena. Examples of the former include nesting fluctuations or peaks in the density of states arising through hot spots [15], turning points [16], or van Hove singularities [17]. These can all be seen as manifestations of impeded cyclotron motion [18] which can result in a linear MR more generically. A recent Boltzmann-based simulation of the in-plane MR

of Nd-LSCO ($p = 0.24$) [17] reproduced the H-linear MR at high fields with the correct slope. The parameterisation for the simulation involved an in-plane anisotropy of the mean-free-path ℓ of order 1000 (attributed to small-q scattering and proximity of the Fermi level to the van Hove saddle-point). Such an extreme parameterisation – though consistent with other studies on 24%-doped Nd-LSCO [17] – is illustrative of the central weakness of such models: they each rely on specific features that are known to vary greatly between the different families, all of which exhibit a H-linear MR of the same magnitude, T-dependence and p-dependence. As explained in the main article, hole-doping Bi2201 and Tl2201 across the SM regime brings the Fermi level closer to, not further away from, the van Hove point (thereby enhancing the in-plane anisotropy in ℓ), yet still the magnitude of the MR drops with increasing p.

If this type of model were to explain the MR of overdoped cuprates, the ubiquity of the MR and its properties would seem to be accidental. We believe this to be unlikely. Further inconsistencies with Boltzmann-based models have been detailed elsewhere [11]. To summarise here for completeness, Boltzmann transport predicts that the magnitude of the MR is largely governed by $\omega_c\tau$ and its anisotropy around the Fermi surface. The variation in τ for example between Tl2201 and Bi2201 as estimated from their residual resistivities is a factor of 10-20 yet the magnitude of the H-linear MR is the same in both cases. In fact, the scaling of the MR with T and not $T + T_0$ shows that the elastic (impurity) component of the scattering rate is not contributing to the MR at all. In the case of Bi2201 where the residual resistivity is a large fraction of the total resistivity, this is particularly constraining for any Boltzmann-based analysis.

The second category of models incorporate real-space inhomogeneity as a key ingredient. Both binary [25] and continuous [26] resistivity distributions [25] using effective medium theory (EMT) as well as those invoking carrier density inhomogeneity [27] (equivalent to EMT modelling) have all been used to generate a H-linear MR in cuprates. These models typically include *ad hoc* a T-linear resistivity having a negligible residual term in order to reproduce H/T scaling. That the resistivity of OD cuprates has an appreciable T^2 component and – especially in the case of Bi2201 – a large residual resistivity ρ_0, these models are unlikely to be more generally applicable. Although not applied to cuprates, continuous mobility distributions modelled using random resistor networks (RNNs) [28] has also been shown to produce a robust H-linear MR but suffers from the same pitfalls in the

reproduction of H/T scaling. A more general argument can be made that the large variations in disorder levels and electronic inhomogeneity present in Bi2201, LSCO and Tl2201 will always make the similarity in form and magnitude of the MR seen in different families or the observed relation to intrinsic properties such as T_c difficult to account for within such models.

Finally, we turn to consider other theoretical proposals that interpret the H-linear and/or T-linear resistive behaviour from different perspectives. A recent proposal by Marino and Arouca, for example, specifically claims to capture the crossover to H-linear MR in the overdoped cuprates [20]. One of the notable features of this model is the presence of a remnant pseudogap within the strange metal regime, whose transition temperature $T^*(x)$ scales with α_1 (the coefficient of the T-linear resistivity) and extends all the way to the end of the SC dome [20]. One of the defining signatures of the opening of the pseudogap in cuprates is a marked decrease in the superfluid density n_s. In Tl2201 [21, 22], LSCO [23] and (to a lesser extent) Bi2201 [24], n_s *increases* with decreasing p right across the strange metal regime, i.e. from the edge of the SC dome all the way to $p^* \sim 0.20$. Hence, one of the defining features of the above model appears to be absent, at least in the three families considered here. Another alternative model assumes that a T-dependent carrier density (also associated with the pseudogap), coupled with a T^2 scattering rate, is essential to the understanding the T-linear resistivity and Hall coefficient R_{H} [19]. While R_{H} in Tl2201 and Bi2201 is weakly T-dependent at low fields (peaked at around 50-100 K), the T-dependence is gradually diminished with increasing field strength, consistent with an anisotropy in ℓ that is progressively washed out. As a result, $R_{\mathrm{H}}(T)$ is essentially T-independent at high-fields, indicating a carrier density that remains constant over the (broad) temperature range of our study. Similarly, the slope of the H-linear MR (once attained) is also found to be T-independent, consistent with a constant carrier density.

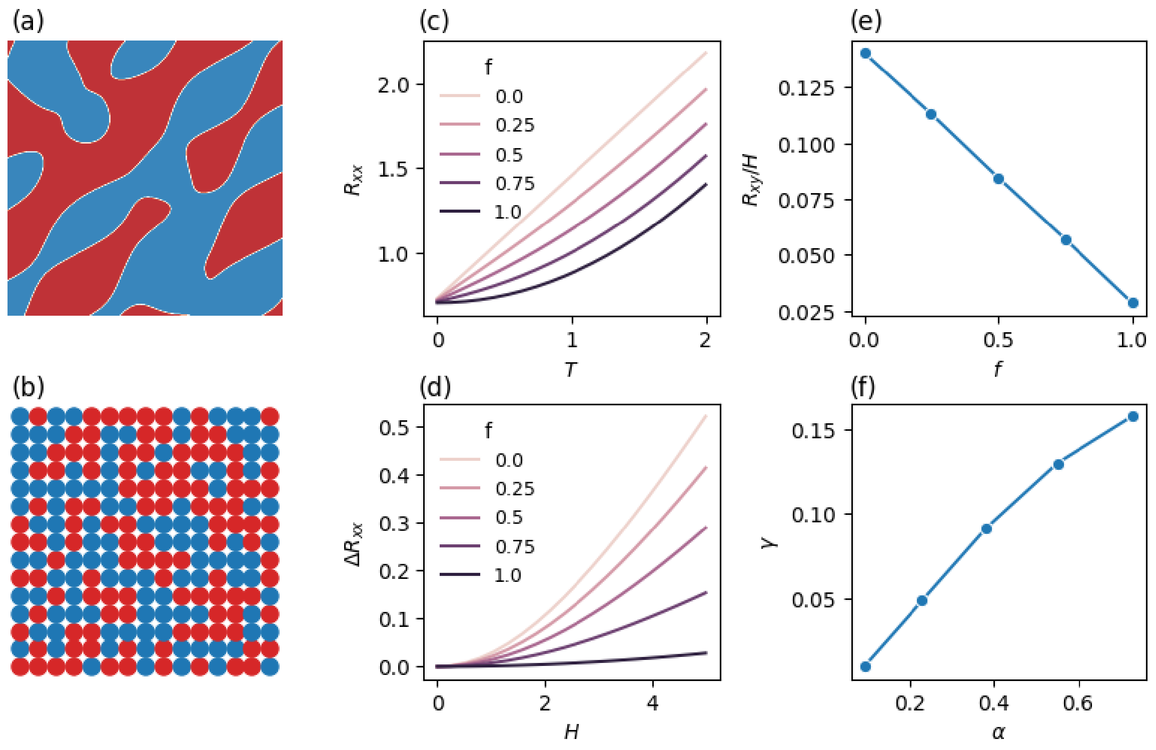

Supplementary Figure 4. Modelling the evolution of the MR with a 4-terminal resistor network. Schematic representation of (a) a sample comprised of two spatially separated carrier types and (b) a corresponding grid-like resistor network comprised of 4-terminal resistor units of two randomly distributed types. The potential and current at each terminal is computed using Kirchhoff's laws. Here, a fraction f of the resistors are assigned $R_{FL} = 1 + 0.25T^2$ and the remainder are assigned $R_{SM} = 1+T$. (c) Zero-field resistance of the network as f is tuned between 0 (SM component only) and 1 (FL component only). A systematic reduction in the T-linear resistivity at $T = 0$ is clearly observed with increasing f. (d) Assuming that the mobility of the SM resistors $\mu_{SM} = 5\mu_{FL}$, $\Delta R(f, T = 0)$ drops markedly as the concentration of FL-like resistors is increased. (e) Similarly, the Hall coefficient ($R_H(T = 0)$) is reduced with increasing f. (f) Both α_1 and γ_1 are found to smoothly increase with an increasing fraction of SM resistors populating the network.

V. RESISTOR NETWORK MODELLING OF THE RESISTIVITY AND MR

As an alternative to the effective medium theory introduced in the main manuscript, one can also use a resistor network comprised of four-terminal resistor units in which each

unit is characterised by a resistance and an effective Hall mobility [40] that generate a local longitudinal and Hall voltage that are to be solved for using Kirchhoff's laws after an arbitrary potential is applied to one end of the sample (e.g. the left hand side of the sample). The macroscopic resistance is then found by summing the input currents along the left edge of the sample and the Hall resistance is found by finding the resultant voltage drop generated between the top and bottom edges. In this case, binary real-space patchiness (as shown schematically in Supplementary Figure 4(a)) can be modelled as a two-dimensional grid (as shown schematically in Supplementary Figure 4(b)) in which a fraction f of the resistor units are assigned FL-like properties and the remainder are assigned SM properties as was done in the main text.

Here, the FL resistors are assigned a resistance $R_{FL} = 1 + 0.25T^2$ and the SM resistors are assigned a resistance $R_{SM} = 1 + T$. As in the case of effective medium theory, this is also sufficient to reproduce a resistivity that at lowest temperatures has the observed $\alpha_1 T + \beta T^2$ form with α_1 decreasing as a smooth function of f (the fraction of FL resistor units) (Supplementary Figure 4(c)). In such a network, the magnitude of both the Hall coefficient and MR are governed by the mobility of its constituent resistors. For example, if the fraction f of FL-like resistors are assigned a lower mobility than the SM resistors at a given temperature, one easily reproduces the smooth reduction in the magnitude of the MR (Supplementary Figure 4(d)) and of R_H (Supplementary Figure 4(e)) that is seen experimentally.

VI. BOLTZMANN SIMULATION OF MR IN OVERDOPED LSCO AND BI2201

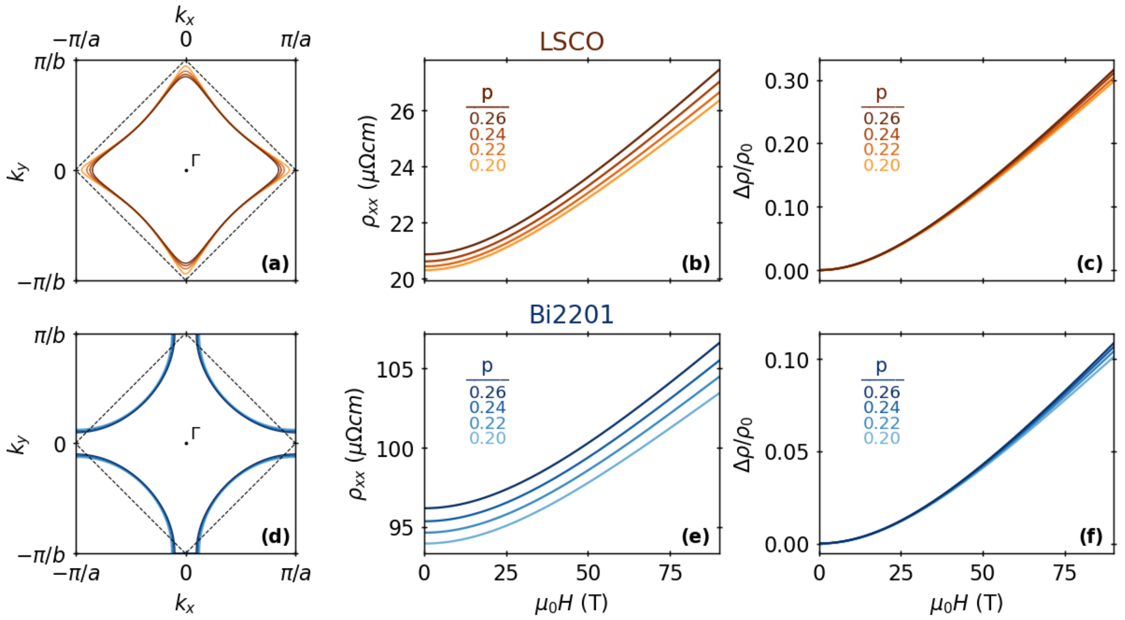

Supplementary Figure 5. (a) Fermi surface of LSCO for $0.20 \leq p \leq 0.26$ as determined from the tight-binding parameterization given in Ref. [29]. (b,c) Corresponding $\rho(H)$ and $\Delta\rho/\rho(0)$ curves at $T = 10$ K derived with the Shockley-Chambers tube-integral formalism of the Boltzmann transport equation assuming a scattering rate anisotropy with the same ϕ-dependence as $1/v_F(\phi)$ derived from panel (a). Despite the marked reduction in anisotropy with increasing p, the MR is essentially unchanged. This is largely due to the fact that the MR is dominated by those carriers with the longest ℓ, i.e. near the zone diagonals and their effective ℓ remains the same across this doping range. (d) Fermi surface of Bi2201 for $0.20 \leq p \leq 0.26$ as determined from the tight-binding parameterization given in Ref. [13]. Note how the Fermi surfaces of LSCO (Bi2201) move, respectively, away from (towards) the saddle points at the zone edges with increasing p. (e,f) Corresponding $\rho(H)$ and $\Delta\rho/\rho(0)$ curves at $T = 10$ K assuming a scattering rate anisotropy with the same ϕ-dependence as $1/v_F(\phi)$ derived from panel (c). In this case, the MR increases slightly with increasing p, due to the fact that the anisotropy around the Fermi surface, which starts out very small at $p = 0.20$, grows rapidly as the Fermi level approaches the van Hove singularity.

---

[1] Presland, M. R. Tallon, J. L. Buckley, R. G. Liu, R. S. & Flower, N. E. General trends in oxygen stoichiometry effects on T_c in Bi and Tl superconductors. *Physica C* **176**, 95 (1991)

[2] Nakamae, S. Behnia, K. Mangkorntong, N. Nohara, M. Takagi, H. Yates, S. J. C. & Hussey, N. E. Erratum: Electronic ground state of heavily overdoped nonsuperconducting $La_{2-x}Sr_xCuO_4$ [Phys. Rev. B 68, 100502 (2003)]. *Phys. Rev. B* **79**, 219904(E) (2009)

[3] Cooper, R. A., Wang, Y., Vignolle, B., Lipscombe, O. J., Hayden, S. M., Tanabe, Y., Adachi, T., Koike, Y., Nohara, M., Takagi, H., Proust, C. & Hussey, N. E. Anomalous criticality in the electrical resistivity of $La_{2-x}Sr_xCuO_4$. *Science.* **323**, 603-607 (2009)

[4] Abrikosov, A. Quantum magnetoresistance. *Phys. Rev. B* **58** 2788-2794 (1998)

[5] Huynh, K. K., Tanabe, Y., Tanigaki, K., Both electron and hole Dirac cone states in $Ba(FeAs)_2$ confirmed by magnetoresistance. *Phys. Rev. Lett.* **106** 217004 (2021)

[6] Pippard, A. Magnetoresistance in metals. (Cambridge University Press,1989)

[7] Feng. Y., Wang. Y., Silevitch, D. M., Yan, J.-Q., Kobayashi, R. Hedo, M., Nakama, T., Onuki, Y., Suslov, A. V., Mihaila, B., Littlewood, P. B., Rosenbaum, T. F., Linear magnetoresistance in the low-field limit in density-wave materials *Proc. Natl. Acad. Sci. (USA)* **116** 11201 (2019)

[8] Hayes, I. M., McDonald, R. D., Breznay, N. P., Helm, T., Moll, P. J. W., Wartenbe, M., Shekhter, A. & Analytis, J. G. Scaling between magnetic field and temperature in the high-temperature superconductor $BaFe_2(As_{1-x}P_x)_2$. *Nat. Phys.* **12** 916-919 (2016)

[9] Giraldo-Gallo, P., Galvis, J. A., Stegen, Z., Modic, K. A., Balakirev, F. F., Betts, J. B., Lian, X., Moir, C., Riggs, S. C., Wu, J., Bollinger, A. T., He, X., Božović, I., Ramshaw, B. J., McDonald, R. D., Boebinger, G. S. & Shekhter, A. Scale-invariant magnetoresistance in a cuprate superconductor. *Science.* **361**, 479-481 (2018)

[10] Ando, Y. & Segawa, K. Magnetoresistance of untwinned $YBa_2Cu_3O_y$ single crystals in a wide range of doping: anomalous hole-doping dependence of the coherence length. *Phys. Rev. Lett.* **88** 167005 (2002)

[11] Ayres, J., Berben, M., Čulo, M., Hsu, Y., Heumen, E., Huang, Y., Zaanen, J., Kondo, T., Takeuchi, T., Cooper, J. R., Putzke, C., Friedemann, S., Carrington, A. & Hussey, N. E. Incoherent transport across the strange metal regime of highly overdoped cuprates. *Nature.* **595** 661-667 (2021)

[12] Ataei, A. & Gourgout, A. & Grissonnanche, G. & Chen, L. & Baglo, J. & Boulanger, M.-E. & Laliberté, F. & Badoux, S. & Doiron-Leyraud, N. & Oliviero, V. & Benhabib, S. & Vignolles, D. & Zhou, J.-S. & Ono, S. & Takagi, H. & Proust, C. & Taillefer, L. Electrons with Planckian scattering obey standard orbital motion in a magnetic field. *Nat. Phys.* **18**, 1420–1424 (2022)

[13] Berben, M., Smit, S., Duffy, C., Hsu, Y., Bawden, L., Heringa, F., Gerritsen, F., Cassanelli, S., Feng, X., Bron, S., Heumen, E., Huang, Y., Bertran, F., Kim, T., Cacho, C., Carrington, A., Golden, M. & Hussey, N. E. On the superconducting dome and pseudogap endpoint in Bi2201. *Phys. Rev. Mater.* **6** 044804 (2022)

[14] Naito, M. & Tanaka, S. Galvanomagnetic effects in the charge-density-wave state of 2H-$NbSe_2$ and 2H-$TaSe_2$. *J. Phys. Soc. Japan.* **51** 228-232 (1982)

[15] Koshelev, A. E. Magnetotransport of multiple-band nearly antiferromagnetic metals due to hot-spot scattering. *Phys. Rev. B* **94** 125154 (2016)

[16] Koshelev, A. E. Linear magnetoconductivity in multiband spin-density-wave metals with non-ideal nesting. *Phys. Rev. B* **88** 060412(R) (2013)

[17] Grissonnanche, G., Fang, Y., Legros, A., Verret, S., Laliberté, F., Collignon, C., Zhou, J., Graf, D., Goddard, P. A., Taillefer, L. & Ramshaw, B. J. Linear-in temperature resistivity from an isotropic Planckian scattering rate. *Nature* **595** 667-672 (2021)

[18] Hinlopen, R. D. H., Ayres, J., Hinlopen, F. A. & Hussey, N. E. B^2 to B-linear magnetoresistance due to impeded orbital motion. *Phys. Rev. Res.* **4** 033195 (2022)

[19] Barišić, N., Chan, M. K., Veit, M. J., Dorow, C. J., Ge, Y., Li, Y., Tabis, W., Tang, Y., Yu, G., Zhao, X., Greven, M. Evidence for a universal Fermi-liquid scattering rate throughout the phase diagram of copper-oxide superconductors *New J. Phys.* **21** 113007 (2019)

[20] Marino, E. C., Arouca, R., Magnetic field effects on the transport properties of high-T_c cuprates *Supercond. Sci. and Tech.* **34** 085008 (2021)

[21] Uemura, Y. J. and Keren, A. and Le, L. P. and Luke, G. M. and Wu, W. D. and Kubo, Y. and Manako, T. and Shimakawa, Y. and Subramanian, R. and Cobb, J. L. and Markert, J. T. Magnetic-field penetration depth in $Tl_2Ba_2CuO_{6+\delta}$ in the overdoped regime, *Nature* **364**, 605-607 (1993)

[22] Niedermayer, Ch. and Bernhard, C. and Binninger, U. and Glückler, H. and Tallon, J. L. and Ansaldo, E. J. and Budnick, J. I. Muon spin rotation study of the correlation between T_c and n_s/m^* in overdoped $Tl_2Ba_2CuO_{6+\delta}$, *Phys. Rev. Lett.* **71**, 1764—1767 (1993)

[23] Božović, I. and He, X. and Wu, J. and Bollinger, A. T. Dependence of the critical temperature in overdoped copper oxides on superfluid density, *Nature* **510**, 309-311 (2016)

[24] Tromp, W. O., Benschop, T., Ge, J.-F., Battisti, I., Bastiaans, K. M., Chatzopoulos, D., Vervloet, A. H. M., Smit, S., van Heumen, E., Golden, M. S., Huang, Y., Kondo, T., Takeuchi, T., Yin, Y., Hoffman, J. E., Sulangi, M. A., Zaanen, J. & Allan, M. P.. Puddle formation and persistent gaps across the non-mean-field breakdown of superconductivity in overdoped $(Pb,Bi)_2Sr_2CuO_{6+\delta}$. *Nat. Mat.* **22** 703-708 (2023)

[25] Boyd, C. & Phillips, P. W. Single-parameter scaling in the magnetoresistance of optimally doped $La_{2-x}Sr_xCuO_4$. *Phys. Rev. B* **100** 155139 (2019)

[26] Patel, A. A., McGreevy, J., Arovas, D. P. & Sachdev, S. Magnetotransport in a model of a disordered strange metal. *Phys. Rev. X* **8** 021049 (2018)

[27] Singleton, J. Temperature scaling behavior of the linear magnetoresistance observed in high-temperature superconductors. *Phys. Rev. Mat.* **4** 061801(R) (2020)

[28] Parish, M. J. & Littlewood, P. B. Non-saturating magnetoresistance in heavily disordered semiconductors. *Nature* **426** 162-165 (2003)

[29] Yoshida, T., Zhou, X. J., Lu, D. H., Komiya, s., Ando, Y., Eisaki, H., Kakeshita, T., Uchida, S., Hussain, Z., Shen, Z.-X. & Fujimori, A. Low-energy electronic structure of the high-T_c cuprates $La_{2-x}Sr_xCuO_4$ studied by angle-resolved photoemission spectroscopy. *J. Phys.: Condens. Matt.* **19** 125209 (2007)
